# Incidence and risk of attention‐deficit hyperactivity disorder in children with amblyopia: A nationwide cohort study

**DOI:** 10.1111/ceo.13465

**Published:** 2019-02-14

**Authors:** Chien‐Chia Su, Chia‐Ying Tsai, Tzu‐Hsun Tsai, I‐Ju Tsai

**Affiliations:** ^1^ Department of Ophthalmology National Taiwan University Hospital, National Taiwan University Taipei Taiwan; ^2^ Graduate Institute of Clinical Medicine, College of Medicine National Taiwan University Taipei Taiwan; ^3^ Department of Ophthalmology Fu Jen Catholic University Hospital, Fu Jen Catholic University New Taipei City Taiwan

**Keywords:** amblyopia, attention‐deficit hyperactivity disorder, children, incidence

## Abstract

**Importance:**

The association between visual deficits and attention disorders has been reported but remains unproven.

**Background:**

The objective of this study was to evaluate the risk of attention‐deficit hyperactivity disorder (ADHD) in children with amblyopia.

**Design:**

Population‐based, cohort study.

**Participants:**

The dataset from the Taiwan National Health Insurance Research Database in 2000 to 2010.

**Methods:**

A total of 6817 patients aged <18 years with newly diagnosed amblyopia were identified. Four age‐ and sex‐matched controls without amblyopia were included for each patient, that is, 27268 controls.

**Main Outcome Measures:**

The primary outcome was the risk of ADHD. The secondary outcomes were age at ADHD onset and use of ADHD medication.

**Results:**

During a mean observation period of 7.18 years, the incidence of ADHD per 1000 person‐years was 7.02 in the amblyopia group and 4.61 in the control group (*P* < 0.0001). The ADHD risk in the amblyopia group was 1.81 times that in the control group (hazard ratio 1.81; 95% confidence interval 1.59‐2.06). After stratification by amblyopia subtype, the greatest risk was in the deprivation type (hazard ratio 2.14; 95% confidence interval 1.56‐2.92) followed by the strabismic (hazard ratio 2.09; 95% confidence interval 1.15‐3.79) and refractive (hazard ratio 1.76; 95% confidence interval 1.54‐2.02) types. Age at ADHD onset was younger in the amblyopia group (median 8.14 vs 8.45 years; *P* = 0.0096). The average duration of neuropsychiatric medication use was comparable between groups (*P* = 0.98).

**Conclusions and Relevance:**

The ADHD risk is higher in children with amblyopia.

## 1 INTRODUCTION

Amblyopia is a common cause of vision impairment and has prevalence rates of approximately 2% in preschool children and 3% in adult population.^1^, ^2^ It is characterized by impaired vision in one or both eyes because of disruption of normal visual stimuli and underdevelopment of the visual cortex because of strabismus, pattern deprivation or inadequate refractive error correction during the critical period.^3^ Recent studies have demonstrated that abnormal visual experience may influence the microstructural integrity of the white matter and cortical changes.^4^, ^5^

Attention‐deficit hyperactivity disorder (ADHD) is one of the most prevalent neurodevelopmental disorders in children, with an overall estimated prevalence of 7.2% in pooled school and community populations.^6^ The clinical presentation includes inattentiveness, hyperactivity, impulsivity and cognitive deficits. Symptoms such as inattentiveness and hyperactivity‐impulsivity appear during childhood and interfere with social and academic functioning in affected individuals. ADHD is a heterogeneous disorder of unknown aetiology and the diagnosis generally requires careful psychiatric interviews and assessment of various aspects of the child's life. Cortical abnormalities, decreased integrity of the white matter and disturbed structural connectivity have been demonstrated in patients with ADHD.^7^, ^8^

Some studies have reported an association between visual deficits and attention disorders. One study showed that certain ophthalmic disorders, such as amblyopia, cataract and nystagmus, could affect the visuo‐attentional performance of children aged 4‐7 years.^9^ Gronlund et al reported frequent occurrence of various ophthalmic disorders, including subnormal visual acuity, heterophoria, refractive errors and morphological anomalies in the retinal vasculature and optic nerve in children with ADHD.^10^ The authors have speculated that inattentiveness may be attributed to poor visual acuity and that a common neurodevelopmental problem likely precedes the ophthalmic problems and ADHD. Despite increasing evidence of an association between vision problems and ADHD, it has been difficult to reach a conclusion regarding this association because of small sample sizes, limited representativeness of study subjects, and/or the lack of a case‐control design in previous studies.

Visual acuity screening is compulsory for all preschool children at 4 years of age and an annual check of visual acuity is required in all school‐aged children in Taiwan. Any child who fails the vision screen is referred for a comprehensive ophthalmic evaluation. The National Health Insurance program in Taiwan is a mandatory general health insurance program that covers 99% of the 23 million individuals residing in Taiwan. To overcome the limitations of previous studies, we used this database to gather a large cohort for the present study, the aim of which was to compare the incidence and risk of ADHD between children with amblyopia and those without amblyopia.

## 2 METHODS

We conducted a retrospective population‐based cohort study using the Longitudinal Health Insurance Database of Taiwan's National Health Insurance Research Database, which includes a million beneficiaries randomly selected from all insurers in the National Health Insurance program. The research protocol was approved by the Ethics Review Board of the National Taiwan University Hospital, Taipei, Taiwan.

The study included patients aged <18 years who were newly diagnosed with amblyopia between 2000 and 2010. Patients with a history of neuropsychiatric medication intake and/or those with a previous diagnosis of ADHD before the study selection period were excluded. We also identified and recruited four age‐ and sex‐matched children without a diagnosis of amblyopia as controls for each patient.

In clinical practice, children with suboptimal corrected visual acuity in one or both eyes due to disruption of visual stimuli were defined as amblyopes and coded as having amblyopia in the national health insurance system. The children were diagnosed with amblyopia on the basis of the amblyopia diagnostic codes 368.00, 368.01, 368.02 or 368.03 (International Classification of Diseases, Ninth Revision, Clinical Modification [ICD‐9‐CM]) and the findings of visual acuity examinations. Patients with amblyopia were further stratified by subtype of amblyopia as strabismic, deprivation or refractive. The deprivation subtype was characterized by the presence of certain ocular anomalies obscurating the visual axis, such as cataract, ptosis or corneal opacity. The strabismic subtype was characterized by the presence of esotropia or exotropia. Cases with mixed diagnostic codes of both deprivation and strabismic subtypes were classified into deprivation subtypes. Cases that could not be categorized as the deprivation or strabismus subtype were categorized as the refractive subtype. All subjects were followed from the index date until the date of death, date of loss to follow‐up (withdrawal from the insurance program) or the date of the last follow‐up.

The primary outcome in the study was the occurrence of ADHD. The diagnosis of ADHD was based on the ICD‐9‐CM codes 314.00, 314.01, 314.1, 314.2, 314.8 or 314.9. With regard to comorbidities, we collected data pertaining to delayed development, mental retardation, autism spectrum disorder, Tourette syndrome, paralysis, epilepsy, asthma, allergic rhinitis and allergic conjunctivitis.

The secondary outcome was the age at onset of ADHD and the duration of neuropsychiatric medications use, including methylphenidate, atomoxetine and bupropion, in patients with ADHD according to the presence or absence of amblyopia.

### 2.1 Statistical analysis

Differences in demographics and comorbidities between the amblyopia group and the control group were examined using the *χ* ^2^ or Fisher's exact test for categorical variables and the Wilcoxon two‐sample or Brown‐Mood test for continuous variables. The cumulative incidence of ADHD was estimated using the Kaplan‐Meier method. A Cox proportional hazards model was used to estimate the hazard ratios (HRs) and 95% confidence intervals (CIs) for the risk of ADHD in both study groups. Baseline characteristics which significantly associated with ADHD in the univariate analysis were included in a multivariate regression model for adjustment. All statistical analyses were performed using SAS version 9.1 software (SAS Institute, Inc., Cary, North Carolina). A *P* value <0.05 was considered statistically significant.

## 3 RESULTS

**Figure 1:**
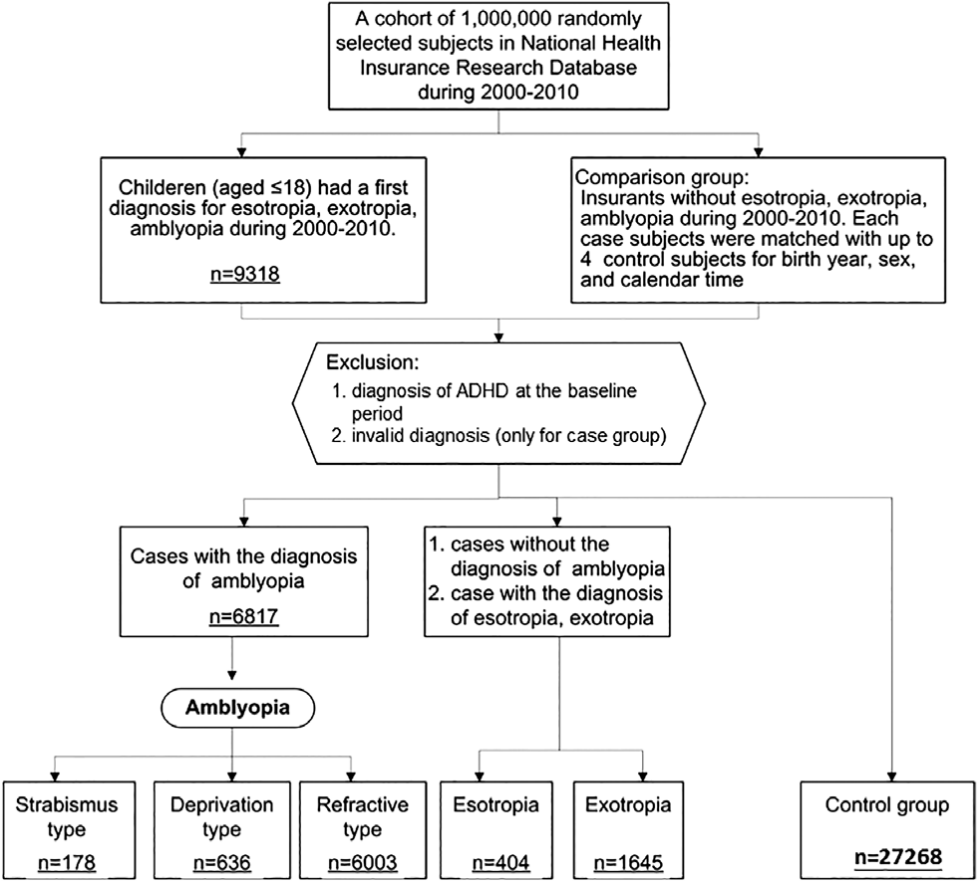
Flowchart showing selection of patients for the study from the National Health Insurance Research Database in Taiwan. Amblyopia: ICD‐9‐CM 368.00, 368.01, 368.02 or 368.03; attention‐deficit hyperactivity disorder (ADHD): 314.00, 314.01, 314.1, 314.2, 314.8 or 314.9

In total, 6817 children diagnosed with amblyopia and 27268 controls matched for age and sex were recruited for this study. The flowchart for the study is shown in Figure 1. In the amblyopia group, there were 178 children with the strabismic type, 636 with the deprivation type, and 6003 with the refractive type. Four hundred and four cases of esotropia and 1645 cases of exotropia without a diagnosis of amblyopia were not included in the study.

The mean age of the study subjects was 7.0 years (standard deviation (SD) 2.8 years), and 52.6% were female. Comorbidities, including delayed development, mental retardation, paralysis, epilepsy, asthma, allergic rhinitis and other allergic diseases, was more common in the amblyopia group than in the control group (Table 1).

**Table 1:** Characteristics of patients with amblyopia (cases) and subjects without amblyopia (controls)

Variable	Amblyopia n = 6817	Subtype			Control group n = 27268	*P* value
		Strabismus n = 178	Deprivation n = 636	Refractive n = 6003		
Age (year)						
Mean (SD)	7.0 (2.8)	7.2 (3.3)	6.8 (3.2)	7.0 (2.7)	7.0 (2.8)	0.9047
Female sex, n (%)	3586 (52.6)	77 (43.3)	342 (53.8)	3167 (52.8)	14 344 (52.6)	1
Duration of follow‐up (year)						
Mean (SD)	7.1 (2.6)	7.6 (2.7)	7.2 (2.7)	7.1 (2.6)	7.2 (2.6)	
Comorbiditities, n (%)						
Autism spectrum disorder	9 (0.1)	0 (0)	3 (0.5)	6 (0.1)	7 (0.0)	
Tourette syndrome	1 (<0.01)	0 (0)	0 (0)	1 (0.0)	4 (0.0)	
Delayed development	115 (1.7)	2 (1.1)	26 (4.1)	87 (1.4)	76 (0.3)	<0.0001
Mental retardation	32 (0.5)	1 (0.6)	6 (0.9)	25 (0.4)	16 (0.1)	<0.0001
Paralysis	57 (0.8)	2 (1.1)	23 (3.6)	32 (0.5)	17 (0.1)	<0.0001
Epilepsy	46 (0.7)	1 (0.6)	12 (1.9)	33 (0.5)	38 (0.1)	<0.0001
Lower respiratory tract infection	2685 (39.4)	73 (41.0)	259 (40.7)	2353 (39.2)	4876 (17.9)	<0.0001
Asthma	476 (7.0)	10 (5.6)	35 (5.5)	431 (7.2)	760 (2.8)	<0.0001
Allergic rhinitis	770 (11.3)	13 (7.3)	58 (9.1)	699 (11.6)	1336 (4.9)	<0.0001
Allergic conjunctivitis	296 (4.3)	8 (4.5)	26 (4.1)	262 (4.4)	380 (1.4)	<0.0001

The mean observation period was 7.18 years. The incidence of ADHD per 1000 person‐years was 7.02 and 4.61, respectively, in the amblyopia and control groups (Table 2). Multivariate Cox regression analysis showed that the risk of ADHD in the amblyopia group was 1.8 times that in the control group (95% CI 1.59‐2.06) after adjustment for age, sex and medical comorbidities. All three subtypes of amblyopia were associated with an increased risk of ADHD; the adjusted HRs for the deprivation, strabismic and refractive subtypes were 2.14 (95% CI 1.56‐2.92), 2.09 (95% CI 1.15‐3.79) and 1.76 (95% CI 1.54‐2.02), respectively.

**Table 2:** Hazard ratios for amblyopic children among all sampled subjects^a^

				Hazard ratio (95% CI)			
	Person year	ADHD events	Incidence^b^	Crude	Adjusted	Crude *P* value	Adjusted *P* value
Comparison group	196190	905	4.61	1 (reference)	1 (reference)		
Patients with amblyopia	48711	342	7.02	1.52(1.35,1.73)	1.81(1.59, 2.06)	<0.0001	<0.0001
Refractive	42804	288	6.73	1.46(1.28, 1.66)	1.76(1.54, 2.02)	<0.0001	<0.0001
Strabismus	1344	11	8.18	1.82(1.01, 3.31)	2.09(1.15, 3.79)	<0.0001	0.0153
Deprivation	4563	43	9.42	2.06(1.51, 2.79.) Trend Test	2.14(1.56, 2.92.) P < 0.0001	<0.0001	<0.0001

a Models adjusted for age, sex, delayed development, mental retardation, lower respiratory tract infection and paralysis. These variables were significant in the univariate logistic regression analysis.

b Per 1000 person‐years.

**Figure 2:**
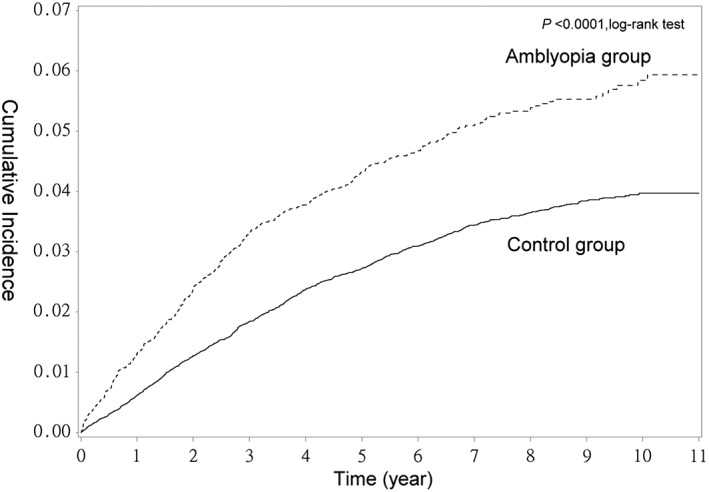
Cumulative incidence of attention‐deficit hyperactivity disorder (ADHD) in the amblyopia group and the control group

The cumulative incidence of ADHD was significantly greater in the amblyopia group than in the control group (Figure 2, *P* < 0.0001, log‐rank test).

The median age at onset of ADHD was younger in children with amblyopia than in those without amblyopia (8.14 years vs 8.45 years; *P* = 0.0096; Table 3). The average duration of neuropsychiatric medications use was comparable between the amblyopia and control groups (10.98 months vs 10.15 months; *P* = 0.98). The durations of treatment with methylphenidate (*P* = 0.97), atomoxetine (*P* = 0.39) and bupropion (*P* = 0.39) were similar between the study groups.

**Table 3:** Characteristics of ADHD patients in children with amblyopia and control group

	Amblyopia n = 342	Control group n = 905	*P* value
Onset age of ADHD	8.49 ± 2.53	8.91 ± 2.60	
Mean (y)	8.49	8.91	
Median (y)	8.14	8.45	0.0096
Medication possession			
Methylphenidate			
Person	192	549	
Month	11.51 ± 15.83	10.76 ± 16.49	0.967
Atomoxetine			
Person	11	42	
Month	5.37 ± 6.73	5.29 ± 5.11	0.386
Bupropion			
Person	6	19	
Month	4.24 ± 3.20	3.41 ± 3.88	0.390
All medications			
Person	209	610	0.982
Month	10.98 ± 15.36	10.15 ± 15.82	

## 4 DISCUSSION

In this population‐based cohort study of data from a nationwide health insurance research database, we demonstrated that children with amblyopia have a greater risk of developing ADHD than their counterparts without amblyopia; moreover, children with amblyopia who developed ADHD tend to be diagnosed at a younger age than those without amblyopia.

Our primary targets in this study were children with amblyopia, including the strabismic type (n = 178), deprivation type (n = 636) and refractive type (n = 6003). Four hundred and four cases of esotropia and 1645 cases of exotropia without monocular visual acuity deficit were not included in our analysis. The distribution of amblyopia subtypes in the present study was similar to that in a previous study of 5232 children in Eastern Taiwan^11^ in which amblyopia was related to strabismus (2.6%), refractive error (62.6%), anisometropia (24.3%) and deprivation (10.4%). Given that vertical strabismus has a relative lower prevalence in strabismic children and is usually accompanied by neurological problems such as cranial nerve palsy, we did not include vertical strabismus as a strabismic subtype. Accordingly, the number of strabismic amblyopes might have been underestimated in our study.

Strabismic amblyopia has also been reported to be uncommon in children from other East Asian countries, including Singapore (15%) and Korea (12.8%).^12^, ^13^ Studies from Asia and the United States suggest that intermittent exotropia is the most prevalent subtype of exotropia,^14^, ^15^ and intermittent exotropia accounted for 51.7% of cases of exotropia in a study of patients aged <19 years.^14^ Interestingly, relatively large numbers of strabismic amblyopes were reported in studies outside of Asia, with 38% of amblyopic children in North America^16^ and 19% of amblyopic adults in Australia^1^ having the strabismic subtype. In addition to exclusion of vertical strabismus, the low amblyogenic risk of intermittent exotropia may be another reason for the smaller proportion of strabismic cases in our study.

In the present study, the incidence of ADHD in children over 10 years of age was significantly higher in the amblyopia group than in the control group (6% vs 3%; *P* < 0.0001, log‐rank test). After adjustment for age, sex, and comorbidities, the presence of amblyopia was found to be associated with a 1.81‐fold increased risk of ADHD. With regard to the different underlying causes of amblyopia, the risk of ADHD was found to be highest in children with the deprivation type followed by those with the strabismic type. Children with refractive amblyopia had the lowest risk (*P* < 0.0001, trend test). Interestingly, this finding was compatible with previously reported visual prognoses for amblyopic eyes with different subtypes, with deprivation amblyopia showing the worst prognosis and refractive amblyopia showing the best.^1^

The potential for a causal relationship between development of ADHD and amblyopia has not been elucidated. We propose here some explanations for the higher incidence of ADHD in children with amblyopia. First, ADHD is a multifactorial disease with some known risk factors, including maternal and paternal smoking during pregnancy, a low birth weight, a high‐blood lead level and a family history of ADHD.^17^, ^18^ It is possible that ADHD and amblyopia share one or more predisposing factors, although we had adjusted for known comorbidities in our regression analysis. The rates of certain comorbidities, including delayed development, mental retardation and respiratory allergic diseases, were higher in the amblyopia group. It is noteworthy that ADHD may be statistically overrepresented among individuals with developmental intellectual disabilities.^19^ Moreover, allergic reactions have been proposed to foster an imbalance in cholinergic/adrenergic activity within the central nervous system, leading to ADHD behaviours in some children, and were reported to independently and synergistically contribute to a higher risk of ADHD in children.^20^ These potential confounders were all adjusted for in the present study.

Second, vision plays an important role in the acquisition of information. Previous studies have reported an association between attention‐deficits and vision disorders.^9^, ^10^ In one national telephone survey that included 75171 US children without intellectual impairment (age 4‐17 years), those with non‐refractive vision problems recognized by vision‐specific questions showed a higher prevalence of ADHD (15.6%) than did those with normal vision (8.3%). Vision problems remained a risk factor (odds ratio 1.8; 95% CI 1.2‐2.7) for current ADHD after adjustment for confounding variables, which is compatible with our findings.^21^

Neuroimaging modalities have been used as investigative tools to study the neuromechanisms of amblyopia and ADHD.^22^ In humans with amblyopia, functional magnetic resonance imaging (MRI) showed not only abnormal activation in a circumscribed part of the V1 region but also extensive extrastriate areas.^22^ Wang et al found that patients with anisometropic amblyopia have abnormal functional connectivity density, which suggests that chronically poor visual input not only impairs the short‐range functional connections in the visual pathways but also affects the long‐range functional connections in the posterior parietal and frontal cortices. These regions subserve visuospatial attention as well as visuomotor and vision‐guided actions.^23^ Another study that investigated white matter changes in children with anisometropic amblyopia found a significant decrease in the fractional anisotropy value for the right optic radiation, left inferior longitudinal fasciculus and right superior longitudinal fasciculus.^24^ Of note, diffusion tensor imaging parameters for the right superior longitudinal fasciculus have been reported to correlate with measures of attention in patients with ADHD.^7^ We speculate that abnormal visual processing may be related to microstructural changes in the brain that may further predispose the individual to attention deficits. However, without assessing behavioural parameters, visual function and structural MRI data in the same patients, any attempt at drawing conclusions regarding the causal relationship must remain speculative.

In the present study, we calculated the duration of prescribed neuropsychiatric medications, including methylphenidate, atomoxetine and bupropion, in children who had ADHD with and without amblyopia. Psychosocial interventions, such as parental training in child behaviour and teacher training in classroom management, are recommended as first‐line management for children with mild to moderate ADHD, whereas stimulants and other medication are recommended for children with more severe ADHD.^25^ In the present study, 133 (38.89%) of 342 children with amblyopia and 295 (32.60%) of 905 children without amblyopia did not receive any ADHD medication. Compatible with general practice guidelines,^25^ methylphenidate was the most commonly prescribed medication in our study; 192 (56.14%) children with amblyopia and 549 (60.66%) without amblyopia had received this medication. The average duration of treatment with methylphenidate did not differ between the groups (*P* = 0.967).

This study has some limitations. First, the diagnoses of amblyopia, ADHD and other comorbid diseases were entirely based on ICD‐9‐CM codes. The database did not include the severity of amblyopia or objective measures of visual acuity and refraction. We could only use the different subtypes of amblyopia as surrogates for the levels of visual performance. Second, there may have been instances where the subtype of amblyopia was misidentified. For example, we identified ptosis as deprivational amblyopia; however, ptosis may also cause refractive or strabismus amblyopia. Nevertheless, the findings of our study demonstrate the risk of development of ADHD in children with amblyopia. Our findings suggest that suboptimal development of visual acuity is associated with an increased risk of ADHD in children with amblyopia. Additional research effort should be directed towards longitudinal studies examining the functional correlation between severity of visual impairment and attention‐deficit and the mechanisms underlying the association between amblyopia and ADHD.

## CONFLICTS OF INTEREST

None declared.
